# From the Turing Test to AI detectors: an epistemological mismatch in scholarly publishing

**DOI:** 10.1016/j.bjane.2026.844740

**Published:** 2026-03-01

**Authors:** Ahmet Rıdvan Doğan, Ali İrfan Doğan

**Affiliations:** a Sakarya Training and Research Hospital, Department of Anesthesiology, Sakarya, Turkey; b Istanbul Esenyurt University, Department of Management Information Systems, Istanbul, Turkey

*Dear Editor,*

Alan Turing’s “imitation game” proposed that if a machine’s responses were indistinguishable from a human’s, even expert judges could not reliably tell the difference.^1^ In scholarly publishing, however, there has been a shift from such human-based judgments toward automated AI text detectors that claim to identify machine-generated writing. This shift raises fundamental epistemological concerns. Empirical evidence indicates that, in certain experimental settings, both human experts and some current AI detection systems have been shown to perform only marginally better than chance in distinguishing AI-generated text from human prose.^2^ Thus, replacing human judgment with algorithmic detection has not meaningfully improved reliability. Given that advanced AI language models are explicitly optimized to produce human-like text, the assumption that another machine can consistently outperform expert readers appears paradoxical.

AI text detectors do not evaluate meaning; instead, they primarily rely on surface-level linguistic proxies such as stylistic regularity and textual predictability.^3^ Consequently, some AI text detectors have been reported to flag a non-trivial proportion of well-written, formally structured academic texts as AI-generated, particularly in specific disciplinary or evaluative contexts.^4^ Ironically, clarity, coherence, and disciplined academic style ‒ hallmarks of high-quality scholarly writing ‒ may increase the likelihood of false-positive classifications. This issue disproportionately affects authors using formulaic scientific language or writing in a second language. Notably, detectors have misclassified a majority of genuine academic essays by non-native English writers as AI-generated, with false-positive rates exceeding 60% reported in controlled evaluations of non-native English academic writing samples using multiple widely deployed detection tools.^3^ Such findings underscore a fundamental conflict with the indistinguishability principle underlying the Turing Test: if text is genuinely human-like, no simple algorithmic signal can reliably reveal its origin.

The editorial consequences of over-reliance on AI detectors are substantial. False-positive labels risk reputational harm, as allegations of AI authorship are difficult to conclusively refute. Unlike plagiarism, AI-generated content lacks verifiable textual overlap, rendering accusations inherently ambiguous. Experimental studies in specific academic domains have demonstrated that AI detectors may incorrectly flag a significant proportion of authentic journal articles, raising serious concerns about their suitability for editorial decision-making.^4^ Moreover, such practices may undermine fairness and diversity in scholarly communication, disproportionately affecting non-native English authors and certain disciplinary writing styles. Excessive dependence on detector scores may also create perverse incentives, encouraging authors to alter otherwise clear prose to avoid suspicion, while offering false reassurance against genuinely AI-generated submissions that evade detection.

In light of these limitations, AI text detectors should be used, at most, as preliminary screening tools rather than definitive arbiters of authorship. Editorial decisions must not hinge on probabilistic detector outputs alone. Even proponents of these technologies caution against their use as sole evidence due to the persistent risk of false positives.^5^ Human editorial judgment ‒ grounded in contextual evaluation, scholarly coherence, and transparency ‒ remains indispensable. When detector outputs are considered, their role should be clearly disclosed, interpreted in conjunction with human editorial assessment, and accompanied by a transparent process that allows authors to respond to or contest such findings. Ultimately, Turing’s original insight remains instructive: when human and machine outputs become indistinguishable, the solution lies not in increasingly speculative detection, but in clear ethical guidance and balanced editorial oversight. AI detectors may assist the process, but they cannot replace human responsibility in safeguarding scholarly integrity.

For transparency, AI-assisted tools were used only for language editing and structural refinement. All substantive content and arguments were developed by the authors, and no undisclosed AI-generated content or ghostwriting was used.

## Data availability statement

No new data were created or analyzed in this study. Data sharing is not applicable to this article.

## Authors' contributions

Ahmet Rıdvan Doğan: Conceptualization of the manuscript; clinical and editorial perspective; drafting and critical revision.

Ali İrfan Doğan: Conceptual contribution on artificial intelligence, algorithmic and computational aspects; Literature support, and critical revision of the manuscript.

All authors approved the final version of the manuscript.

## Institutional Review Board (IRB) approval

Not applicable. This manuscript is a conceptual Letter to the Editor and does not involve human participants, animals, or identifiable data.

## Study registry

Not applicable.

## Declaration of generative AI in the write process

During the preparation of this manuscript, the author(s) used generative AI and AI-assisted tools to support language editing, structural organization, and refinement of academic phrasing. All content was carefully reviewed, edited, and verified by the author(s), who take full responsibility for the accuracy, originality, and integrity of the manuscript.

## Conflicts of interest

The authors declare no conflicts of interest.
